# The neonatal Fc receptor expression during macrophage differentiation is related to autophagy

**DOI:** 10.3389/fimmu.2022.1054425

**Published:** 2022-11-01

**Authors:** Juliette Lamamy, Anthony Larue, Julie Mariot, Christine Dhommée, Marie-Véronique Demattei, Yves Delneste, Valérie Gouilleux-Gruart

**Affiliations:** ^1^ EA 7501 GICC, Tours University, Tours, France; ^2^ CRCI2NA, SFR ICAT, Inserm, CNRS, Angers and Nantes University, Angers, France; ^3^ Laboratory of Immunology and Allergology, CHU d’Angers, Angers, France; ^4^ Laboratory of Immunology, CHU de Tours, Tours, France

**Keywords:** FcRn, autophagy (macroautophagy), macrophages (M1/M2), differentiation, IgG, cancer biology

## Abstract

The neonatal Fc receptor (FcRn) plays a central role in recycling and biodistributing immunoglobulin G. FcRn is also involved in many physiological immune functions as well as pathological immune responses in cancer or autoimmune diseases. Low levels of FcRn in tumor cells and the microenvironment is associated with poor prognosis in non-small cell lung cancers. Among cells that are present in the tumor microenvironment, macrophages express high levels of FcRn. Macrophages are involved in these pathophysiological contexts by their dual differentiation states of pro- or anti-inflammatory macrophages. However, variations in FcRn protein expression have not been described in macrophage subtypes. In this work, we studied FcRn expression in an *in vitro* model of pro- and anti-inflammatory macrophage differentiation. We demonstrated an inverse relation between FcRn protein and mRNA expression in macrophage populations. Autophagy, which is involved in protein degradation and acquisition of phagocytic function in macrophages, participated in regulating FcRn levels. Intravenous immunoglobulin protected FcRn against autophagosome degradation in anti-inflammatory macrophages. Our data demonstrate that autophagy participates in regulating FcRn expression in pro- and anti-inflammatory macrophages. This finding raises new questions concerning the regulation of FcRn in immune functions.

## Introduction

Beside its role in immunoglobulin G (IgG) recycling and transcytosis, neonatal Fc receptor (FcRn) participates in immune functions such as phagocytosis (1), immune complex (cross)-presentation or elimination (2, 3) and epitope spreading (4, 5), so FcRn is a key player in innate and adaptive immune responses (6, 7). Its participation in the host immune response against both bacteria and viruses and more broadly in protection against infections has led to the definition of novel therapeutics based on the understanding of FcRn biology (7). FcRn is expressed in numerous cell types such as epithelial and endothelial cells and hematopoietic cells (8–10) and therefore is found in many organs (11). It is mainly expressed intracellularly in endosomes (> 90%) and transiently on the cell surface after endosome fusion to the membrane (12, 13). Indeed, only 4% of the FcRn pool is expressed at the surface, with 2% able to be internalized by the cell and 2% not (14).

FcRn expression has long been considered ubiquitous without quantification, but variations in FcRn levels have been described in leucocytes of lupus patients (15), in peripheral blood mononuclear cells of patients with pancreatic ductal adenocarcinoma (16) and in tumor cells leading to metabolic changes (17, 18). In colon (19) and pulmonary cancers (20), low FcRn levels were associated with poor prognosis and disease evolution. Consequently, FcRn has been associated with the anti-tumor immune response (21).

Mechanisms concerning the regulation of FcRn expression have been described at different levels. FcRn is regulated by transcription factors such as STAT and NF-κB (22–24) or Sp1, Sp2, Sp3, c-Fos, c-Jun, YY1, C/EBPβ and C/EBPΔ (25, 26), which interact in distinct regions in the promoter of *FCGRT* gene encoding human FcRn. FcRn is also regulated by DNA methylation of the promoter *via* Zbtb7a and Sp1 factors (27) or by microRNA (*hsa-miR-3181)* (28). Besides these factors, a polymorphic variable number of tandem repeats (VNTR) has been linked to high expression of FcRn when expressed as three repetitions on each allele (29). This 3/3 VNTR polymorphism is related to good IgG recycling in patients under antibody treatment (30, 31). At the cellular level and after transcription factor activation, cytokines such as interferon ɣ (IFN-ɣ) and tumor necrosis factor α (TNF-α) or Toll-like receptor ligands have been shown to up- or downregulate FcRn expression in monocytes and a THP-1 cell line (22, 23), so FcRn is an interesting actor in regulating the immune response.

FcRn is expressed in monocytes/macrophages, which are effector cells engaged at the crossroads between innate and adaptive immune responses (8, 32). Monocyte-derived macrophages are recruited in the context of inflammation (33, 34). Depending on the microenvironment, they polarize into pro-inflammatory macrophages or macrophages involved in the resolution of inflammation and in pro-tumoral responses (35, 36). Granulocyte-macrophage colony-stimulating factor (GM-CSF) and macrophage-CSF (M-CSF) are the main factors involved in pro- or anti-inflammatory macrophage polarization (37). In addition, this polarization is finely tuned by many other cytokines such as IFN-γ for the pro-inflammatory subtype or interleukin 4 (IL-4), IL-10 and IL-13 for the pro-tumoral subtype (36).

Owing to the main role played by cytokines and growth factors in regulating cellular immune responses, this study aimed to determine whether FcRn levels, which can be modulated by cytokines, varies during macrophage differentiation and whether FcRn thus participates in inflammation or the tumor microenvironment development .

## Material and methods

### Blood samples

Blood samples from healthy adult volunteers were collected according to institutional research protection guidelines (agreement no. CA-REC-2019-188) at the *Établissement français du sang*, Centre Val de Loire, France.

### Macrophage differentiation and activation

Peripheral blood mononuclear cells were obtained from blood samples by Ficoll density centrifugation (Eurobio Scientific, France). Monocytes were isolated from peripheral blood mononuclear cells by positive selection with anti-CD14 MicroBeads (Miltenyi Biotech, Germany) according to the manufacturer’s recommendations. Monocytes were cultured in serum-free X-VIVO-15 medium (Lonza, Switzerland) at 1x10^6^ cells/ml. Macrophage subtypes were generated after 6 days in culture medium containing recombinant human GM-CSF or M-CSF (both Miltenyi Biotech) at 60 ng/ml. On day 3, the culture medium was changed to fresh complete medium containing M-CSF or GM-CSF at the same concentration. Intravenous IgG (IVIG; CLAIRYG, LFB Biomédicaments, France) 0.01, 1 or 100 µg/ml was added to macrophage cultures for 6 days as indicated. In autophagy experiments, rapamycin (MP Biomedicals, France) 100 nM was added to the culture medium on day 5 for 24 h. In starvation experiments, cells were washed with phosphate buffered saline (PBS), and RPMI fresh medium was added to macrophages for 4 h. At the end of incubation, chloroquine (Sigma-Aldrich, USA) 15 µM was added for 1 h. On day 6, cells were harvested and used for flow cytometry, RT-qPCR or Western blot experiments.

### Macrophage transfection

Macrophages (1x10^6^ cells/ml) were plated in serum-free X-VIVO-15 medium (Lonza) before transfection on day 3. An amount of 20 μM FcRn siRNA (Origene, USA) or siRNA control (Eurogentec, Belgium) was diluted in 200 μl Opti-MEM (Gibco, France) containing 1 μl Lipofectamine RNAiMAX (Thermo Fisher Scientific, USA). After 20-min incubation at room temperature, 40 nM siRNA complexes were added to cells for 4 h. Then medium was removed and fresh complete medium containing M-CSF or GM-CSF 60 ng/ml was added. Seven independent transfections were performed. FcRn gene extinction efficiency was measured by quantitative real-time PCR (RT-qPCR) and macrophage differentiation markers were studied by flow cytometry.

### Flow cytometry analysis

Labeling was performed in PBS, EDTA 2 mM, fetal calf serum 5% at 4°C in 10^5^ cells. After the Fc receptor blocking step (FcR Blocking Reagent, Miltenyi Biotech), cells were stained with mixtures of monoclonal antibodies at the optimal concentration determined in our laboratory. A first fixation step was performed with 4% paraformaldehyde and a second with FoxP3/Transcription Factor Fixation Buffer (R&D Systems, USA). Intracellular FcRn expression was measured with A488 conjugated-FcRn antibody (Clone #937508 R&D Systems) by using the FoxP3/Transcription Factor Permabilization and Wash Buffer kit (R&D Systems) according to the manufacturer’s instructions. The following antibodies were used: anti-CD14 (PerCP-Vio 700, Miltenyi Biotech), anti-CD163 (PE, Miltenyi Biotech), anti-CD206 (PE-Vio 770, Miltenyi Biotech) and anti-CD16 (APC, Beckman Coulter, USA). Cell viability/apoptosis was detected using the APC Annexin V Apoptosis Detection Kit with 7-AAD (#640930, Biolegend, USA). Fluorescence was measured with a Cytoflex cytometer (Beckman Coulter). Data were analyzed with FlowLogic (Miltenyi Biotech). Flow cytometry analysis was performed in singlicate.

### RT-qPCR

Total RNA was isolated from macrophages by using the NucleoSpin RNA Plus XS kit (Macherey-Nagel, France). RT-qPCR assays involved using the GO Script Reverse Transcription System kit (Promega, USA). Real-time PCR was carried out on the QuantStudio3 (Thermo Fisher Scientific) with a master mix (PowerTrack SYBR Green, Thermo Fisher Scientific) according to the manufacturer’s instructions and with the following primer pairs: Actin forward: ATTGGCAATGAGCGGTTC reverse: CGTGGATGCCACAGGACT, GAPDH forward: GAAGGTGAAGGTCGGAGTCAAC, reverse: CAGAGTTAAAAGCAGCCCTGGT, FCGRT forward: CCCTGGCTTTTCCGTGCTT, reverse: TGACGATTCCCACCACGAG. Each sample was analyzed in triplicate. FcRn mRNA levels were normalized to the geometric mean of Actin and GAPDH mRNA levels. FcRn mRNA expression was then reported according to the expression of our control, consisting of monocytes pooled from 3 healthy donors.

### Cytokine assays

On day 5, macrophages were stimulated with 200 ng/ml lipopolysaccharide (*In vivo*Gen, USA) for 16 h. The supernatants were collected on day 6 to measure cytokine/chemokine concentrations. Concentrations were quantified in duplicate by flow cytometry with the Cytoflex cytometer (Beckman Coulter) and fluorescence-encoded beads (LEGENDplex, BioLegend, USA) within the Human Macrophage/Microglia Panel (#740503, 13-plex) for IL-12p70, TNF-α, IL-6, IL-4, IL-10, IL-1β, arginase, TARC, IL-1RA, IL-12p40, IL-23, IFN-γ, interferon-gamma-inducible protein [IP-10]).

### Western blot analysis

Cells were washed with PBS and lysed in Laemmli buffer (Invitrogen, USA) containing 50 mM DTT. Lysates from 2x10^5^ cells were analysed in singlicate by 15% SDS-PAGE, and proteins were transferred to nitrocellulose membranes. The membranes were blocked with 5% non-fat milk in PBS and incubated with the antibodies anti-FcRn (Santa Cruz Biotechnology, USA sc-271745), anti-ATG5 (Cell Signaling Technology, USA, #12994) and anti-LC3-I/II (Cell Signaling Technology, #4108S). Anti-β-actin (Santa Cruz Biotechnology, #sc-47778) or anti-GAPDH (Santa Cruz Biotechnology, # sc-365062) was a loading control. The membranes were developed with an enhanced chemiluminescence Western blot detection reagent (Thermo Scientific, USA). Uncropped Western blot and replicates are presented as **Supplemental Figure S1 and S2** respectively. Densitometry of the bands was quantified by using ImageJ. FcRn, ATG-5 and LC3-I/II expression was normalized to β-actin or GAPDH expression.

### Statistical analysis

All statistical analyses involved using GraphPad Prism 8.0 (GraphPad Software, USA). Differences between groups were determined by paired two-tailed Wilcoxon test and were considered significant at p < 0.05. Results are expressed as mean ± SD. The level of significance is indicated in the figures as: ns = not significant, ∗p ≤ 0.05 and ∗∗p ≤ 0.01.

## Results

### FcRn mRNA and protein show opposite expression in M-CSF– versus GM-CSF–differentiated macrophages

**Figure 1:**
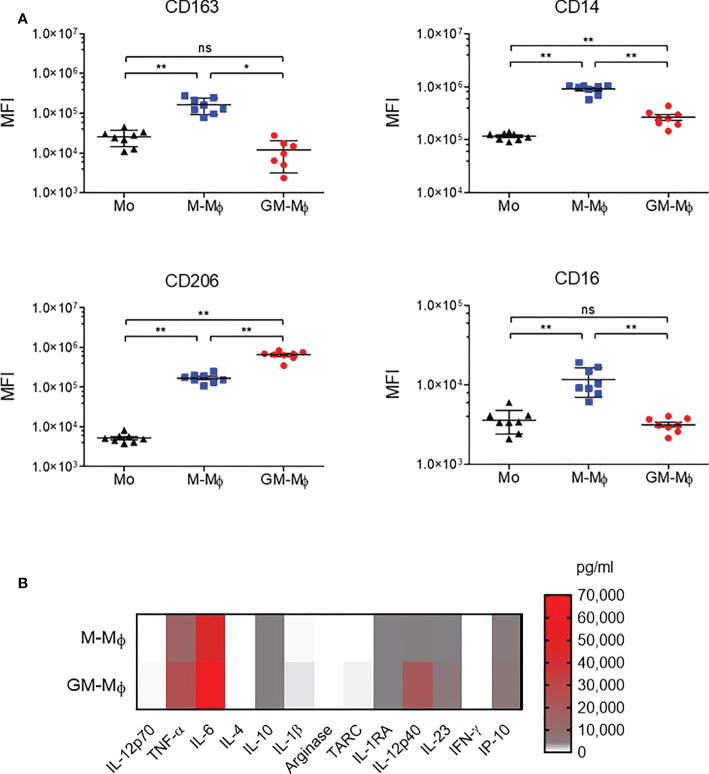
CD163, CD14, CD206 and CD16 analysis by flow cytometry during macrophage differentiation in GM-CSF or M-CSF. Human monocytes were cultured with 60 ng/ml M-CSF or GM-CSF for 6 days. **(A)** CD163, CD14, CD206 and CD16 expression was assessed by flow cytometry on day 0 in monocytes (MO) and day 6 in M-Mϕ and GM-Mϕ. Data are mean fluorescence intensity (MFI) from eight independent experiments (mean ± SD, N=8). **(B)** Results of flow cytometry of cytokine secretion in M-Mϕ and GM-Mϕ after lipopolysaccharide stimulation represented as a heatmap (mean ± SD, N=9). Mo, monocytes; M-Mϕ, M-CSF–induced macrophages; GM-Mϕ, GM-CSF–induced macrophages. ns = not significant, *p ≤ 0.05 and **p ≤ 0.01.

**Figure 2:**
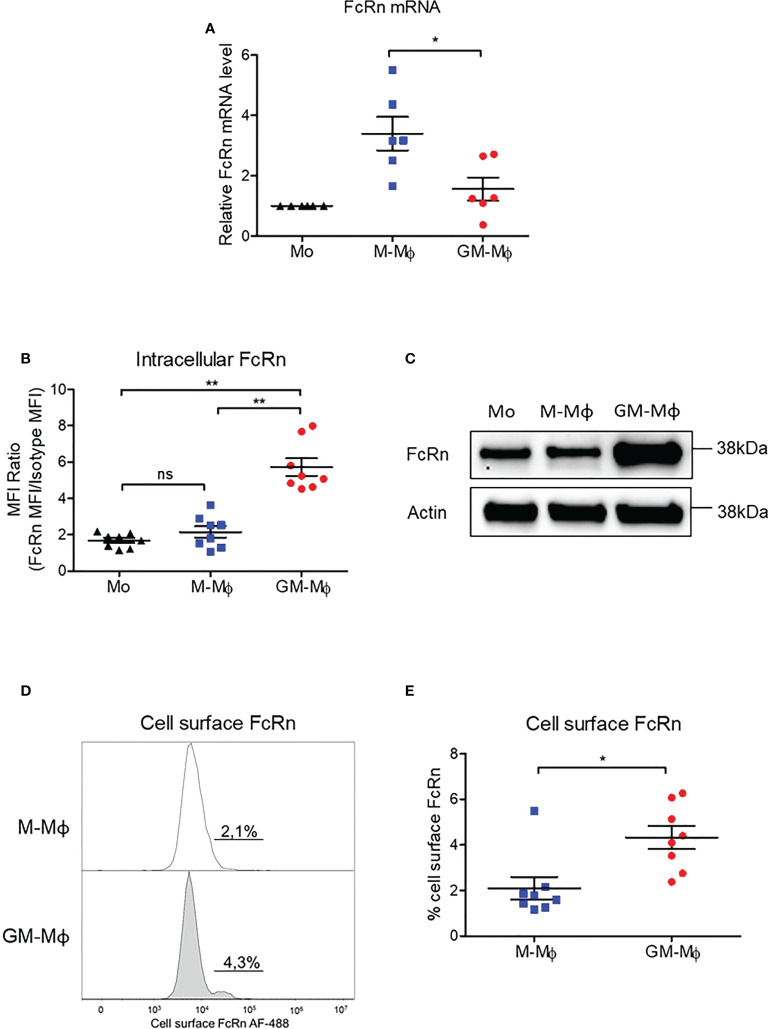
*FCGRT* mRNA and FcRn protein quantification in M-Mϕ and GM-Mϕ. Human monocytes were cultured with 60 ng/ml M-CSF or GM-CSF for 6 days. **(A)** *FCGRT* mRNA was assessed by RT-qPCR and relative expression levels were normalized to *ACTIN* and *GAPDH* mRNA (mean ± SD, N=6). **(B)** Results of flow cytometry of intracellular FcRn expression in monocytes on day 0 and M-Mϕ and GM-Mϕ on day 6. Data are MFI of anti-FcRn monoclonal antibody and isotype MFI ratio (mean ± SD, N=8). **(C)** Western blot analysis of protein extracts purified from monocytes (Mo) on day 0 and M-Mϕ and GM-Mϕ on day 6. **(D)** Flow cytometry of membrane FcRn levels expressed as percentage of positive cells. Histograms of one representative experiment on day 6 is presented for M-Mϕ and GM-Mϕ. **(E)** Mean percentage expression of FcRn cell-surface expression on M-Mϕ and GM-Mϕ (mean ± SD, N=8). Mo, monocytes; M-Mϕ, M-CSF–induced macrophages; GM-Mϕ, GM-CSF–induced macrophages; MFI, mean fluorescence intensity. ns = not significant, *p ≤ 0.05 and **p ≤ 0.01.

Monocytes from healthy donors were purified and cultured for 6 days in the presence of 60 ng/ml M-CSF or GM-CSF to obtain M-CSF macrophages (M-Mϕ) and GM-CSF macrophages (GM-Mϕ), respectively. According to the literature, the expression of CD14, CD163 and CD16 was significantly greater in macrophages after differentiation with M-CSF than GM-CSF, and we also found greater expression of CD206 in GM-Mϕ than M-Mϕ (**Figure 1A**) (38, 39). We also measured IL-12p70, TNF-α, IL-6, IL-4, IL-10, IL-1β, arginase, TARC, IL-1RA, IL-12p40, IL-23, IFN-γ, and IP-10 in culture supernatants of M-Mϕ and GM-Mϕ on day 6 by flow cytometry after lipopolysaccharide stimulation. Consistent with expected data in this differentiation model, levels of pro-inflammatory cytokines such as IL-12, TNF-α and IL-1β were higher in GM-Mϕ than M-Mϕ supernatants (**Figure 1B**). Altogether, CD14, CD163, CD206, CD16 expression markers and cytokine/chemokine profiles attest to consistent macrophage differentiation toward pro- or anti-inflammatory macrophage subtypes. Then, we measured FcRn mRNA by RT-qPCR in differentiated M-Mϕ and GM-Mϕ (**Figure 2A**). FcRn mRNA levels was higher in M-Mϕ than GM-Mϕ and monocytes (**Figure 2A**). In parallel to differentiation markers and FcRn mRNA levels, we measured intracellular FcRn protein levels in monocytes and in M-Mϕ and GM-Mϕ by flow cytometry. In contrast to mRNA analysis, FcRn protein levels were higher in GM-Mϕ than M-Mϕ (mean 5.72 and 2.15) and monocytes (**Figure 2B**). This observation was confirmed in Western blot experiments, with FcRn protein levels greater in GM-Mϕ than M-Mϕ (**Figure 2C**). FcRn protein levels in M-Mϕ and monocytes did not significantly differ. We also examined cell-surface FcRn expression by flow cytometry in M-Mϕ and GM-Mϕ (**Figures 2D, E**). The low levels of FcRn were significantly higher on the GM-Mϕ than M-Mϕ cell surface (4.3% vs 2.1%), according to intracellular FcRn variation.

### Expression of FcRn affects macrophage phenotype

**Figure 3:**
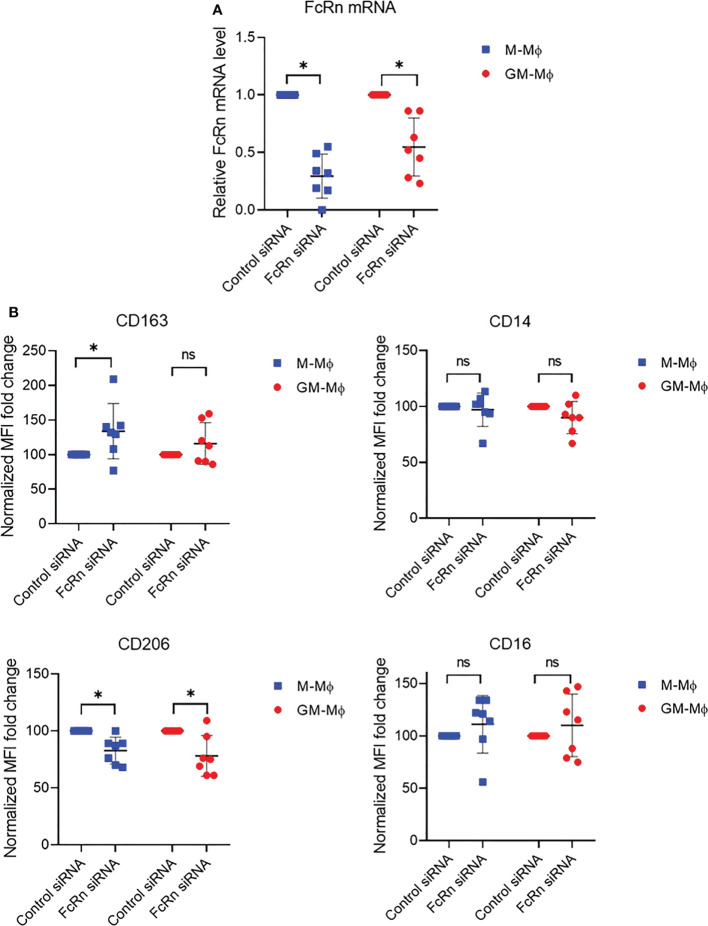
FcRn mRNA and differentiation marker expression after FcRn siRNA transfection. M-Mϕ and GM-Mϕ were transfected with FcRn siRNA on day 3 for 24 h. **(A)** Transfection efficiency was assessed by RT-qPCR. Relative expression of *FCGRT* was normalized to that of actin and GAPDH. Data are relative FcRn levels to siRNA control (mean ± SD, N=7). **(B)** Flow cytometry of CD163, CD14, CD206 and CD16 expression after transfection in M-Mϕ and GM-Mϕ. Data are normalized MFI fold change to siRNA control (mean ± SD, N=7). M-Mϕ, M-CSF–induced macrophages; GM-Mϕ, GM-CSF–induced macrophages; MFI, mean fluorescence intensity; ns, not significant. ns = not significant and *p ≤ 0.05.

To investigate whether FcRn expression could affect macrophage differentiation, we knocked down expression with siRNA at day 3 of culture. We first checked whether monocytes were engaged in differentiation at this time by analyzing the expression of CD163, CD14, CD206 and CD16 markers by flow cytometry. The results confirmed the M-Mϕ and GM-Mϕ phenotypes (**Figure S3A**). FcRn level on day 3 was close to that on day 6, with more FcRn in GM-Mϕ than M-Mϕ (mean 2.61 vs 2.08) (**Figure S3B**). M-Mϕ or GM-Mϕ were then transfected with an FcRn siRNA, which led to a decrease of 70 ± 19% and 45 ± 25% FcRn mRNA levels on M-Mϕ and GM-Mϕ, respectively (**Figure 3A**). The consequence of the decrease in FcRn levels was an increase in CD163 expression (significant in M-Mϕ) and a significant decrease of CD206 expression on M-Mϕ and GM-Mϕ (**Figure 3B**). Neither macrophage subtype showed a significant variation in CD14 and CD16 expression, except for increase, although not significant, for CD16. In total, the reduced FcRn expression in M-Mϕ consolidated their phenotype while turning GM-Mϕ toward the M-Mϕ phenotype.

### Opposite expression of FcRn and autophagy markers in M-Mϕ and GM-Mϕ

**Figure 4:**
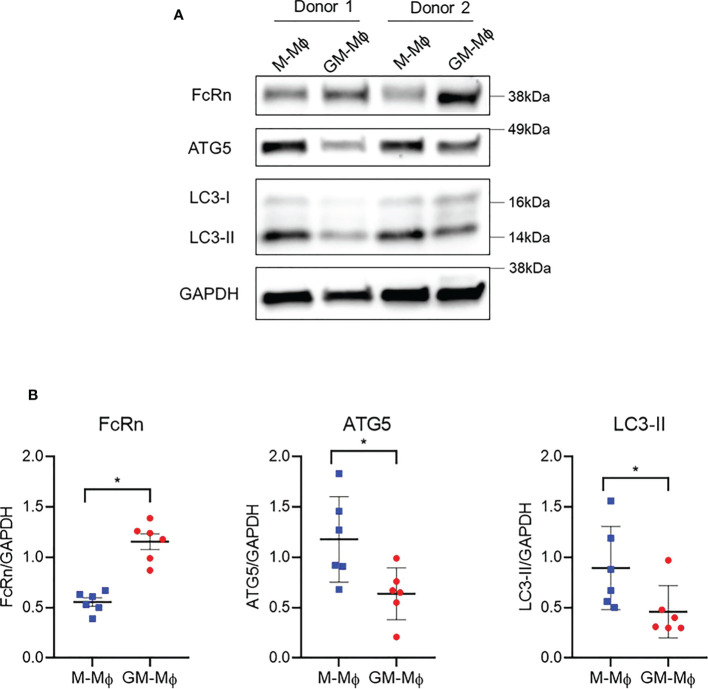
Western blot analysis of protein levels of ATG5, ATG7 and LC3-I/II involved in autophagy and FcRn expression in macrophages. Human monocytes were cultured with 60 ng/ml M-CSF or GM-CSF for 6 days. **(A)** Proteins were extracted from M-Mϕ and GM-Mϕ on day 6. One representative blot is presented. **(B)** Quantification of FcRn, ATG5 and LC3-I/II expression normalized to GAPDH expression (mean ± SD, N=6). M-Mϕ, M-CSF–induced macrophages; GM-Mϕ, GM-CSF–induced macrophages. *p ≤ 0.05.

To investigate the discrepancy between FcRn mRNA and protein levels in M-Mϕ versus GM-Mϕ, we investigated autophagy, a mechanism involved in protein degradation (40). Also, autophagy is a conserved intracellular mechanism required for macrophage polarization and acquisition of phagocytic function (41–43). A recent paper described that FcRn could interact with autophagosomes (44). We examined the expression of autophagy-related 5 (ATG5) and light chain 3 I/II (LC3-I/II) autophagy markers by Western blot analysis (**Figure 4A**). Although FcRn protein levels were, as expected, higher in GM-Mϕ than M-Mϕ, ATG5, which is expressed during the early stage of autophagy, was downregulated in GM-Mϕ versus M-Mϕ. These data suggest higher levels of autophagosome formation in M-Mϕ versus GM-Mϕ. LC3-II protein, widely used to monitor autophagy, followed the same protein level profile as ATG5 (**Figure 4B**), which indicates the presence of a larger number of autophagosomes in M-Mϕ versus GM-Mϕ, opposite to findings for FcRn levels.

### Autophagy regulates FcRn expression in M-Mϕ and GM-Mϕ

**Figure 5:**
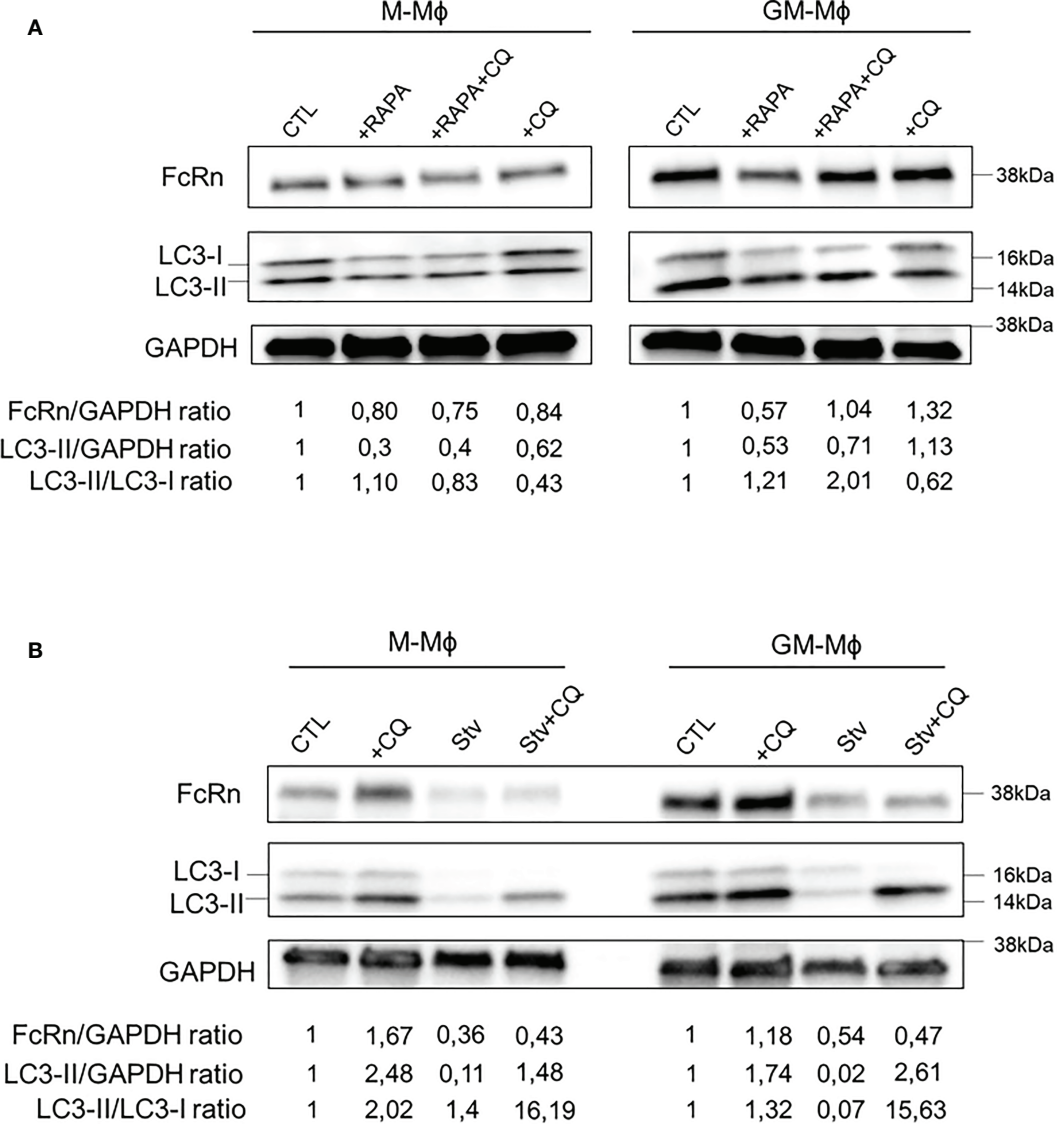
Autophagy activation with rapamycin or starvation modulates FcRn expression. **(A)** M-Mϕ and GM-Mϕ were treated on day 5 with rapamycin for 24 h and with chloroquine for the last hour. **(B)** M-Mϕ and GM-Mϕ were starved on day 6 for 4 h and chloroquine was added for the last hour. In A and B, levels of FcRn and LC3-I/II were examined by Western blot analysis and quantified. Values indicated on the bottom were normalized to GAPDH against the CTL. One representative blot in three experiments is presented for each treatment. M-Mϕ, M-CSF–induced macrophages; GM-Mϕ, GM-CSF–induced macrophages; CTL, control; RAPA, rapamycin (100 nM); CQ, chloroquine (15 µM); Stv, starvation.

We wondered whether FcRn expression was regulated by autophagy. To investigate this, macrophages were incubated on day 5 with or without rapamycin for 24 h to activate autophagy (**Figure 5A**). As indicated, chloroquine was added to culture medium at 1 h before the end of rapamycin treatment to block autophagy flux and quantify FcRn levels and autophagy markers by Western blot analysis. Morphological changes in M- and GM-Mϕ following autophagy treatment are documented in **Figure S4**. Rapamycin incubation for 24 h decreased FcRn levels, which were more pronounced in GM-Mϕ than M-Mϕ. The addition of chloroquine with rapamycin restored FcRn levels in GM-Mϕ. The addition of chloroquine alone, a late-stage inhibitor of autophagy that inhibits autophagosome/lysosome fusion, increased FcRn levels in these cells, with no effect in M-Mϕ. At the same time, LC3-II levels and LC3-II/LC3-I ratios confirmed the autophagy process induced by rapamycin in GM-Mϕ. Another way to activate autophagy process is cell starvation. We applied this treatment to macrophages for 4 h on day 6. FcRn levels were downregulated under starvation conditions with or without chloroquine in both GM-Mϕ and M-Mϕ (**Figure 5B**). As compared with the induction of autophagy by rapamycin, starvation resulted in a total disappearance of LC3-I/II forms, thus reflecting a strong induction of autophagic flux. Moreover, the addition of chloroquine, which allows for the degradation of autophagosomes and thus LC3, confirmed this induction. However, the addition of chloroquine did not rescue FcRn levels under starvation conditions, which suggests an early degradation of the latter from the beginning of the process. Due to the crosstalk between autophagy and apoptosis, we performed Annexin V/7AAD apoptosis analysis after autophagy induction. Rapamycin had no effect on GM-Mϕ apoptosis while it induced autophagy flux and decreased FcRn levels. In M-Mϕ, rapamycin led to 15% of apoptosis coupled to the same consequences on autophagy and FcRn levels. The same effects were observed after starvation even if apoptosis in both macrophage subtypes was more elevated (10% in GM-Mϕ and 20% in M-Mϕ) (**Figure S5**). Chloroquine had no effect on cell apoptosis. These data suggest that apoptosis may not be responsible for FcRn variation levels through autophagy between M- and GM-Mϕ. On flow cytometry after incubation with rapamycin, CD16 expression did not vary, which suggests that the induction of autophagic flux specifically degraded FcRn (**Figure S6**). Altogether, rapamycin and starvation effects attested to the participation of autophagy in regulating FcRn expression.

### FcRn expression and autophagy upregulated by IVIG in M-Mϕ

**Figure 6:**
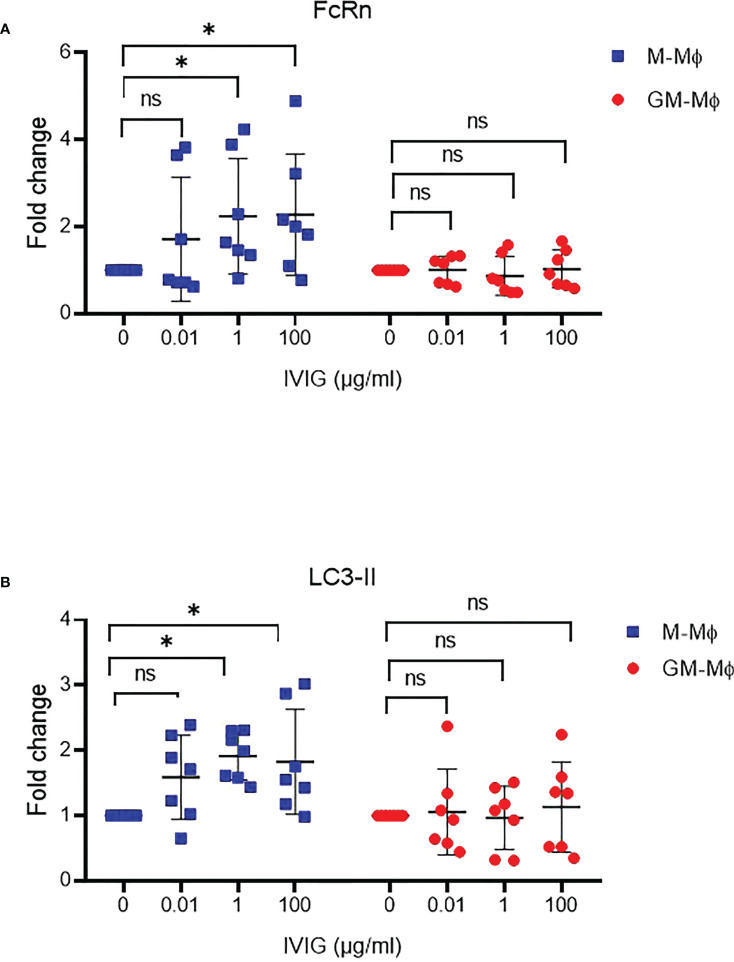
IVIG effect on FcRn expression and autophagy during macrophage differentiation. M-Mϕ and GM-Mϕ were cultured with IVIG 0.1, 1 and 100 µg/ml during differentiation. Western blot analysis of fold change in protein expression for FcRn **(A)** and LC3-II **(B)** quantification and normalization to GAPDH expression (mean ± SD, N=7). M-Mϕ, M-CSF–induced macrophages; GM-Mϕ, GM-CSF–induced macrophages; IVIG, intravenous immunoglobulin G. ns = not significant and *p ≤ 0.05.

IgG is one of the two main ligands of FcRn. Thus, we wondered whether IgG could regulate FcRn expression during macrophage differentiation. Polyclonal IgG 0.01, 1 and 100 µg/ml was added to M- and GM-CSF macrophage cultures from day 0 to day 6. FcRn protein levels were upregulated about 50% in M-Mϕ with 1 and 100 µg/ml IgG but not in GM-Mϕ (**Figure 6A**). This increase in M-Mϕ FcRn levels was associated with elevated LC3-II protein levels, with no significant effect detected in GM-Mϕ (**Figure 6B**), which again suggests a link between FcRn levels and autophagy.

## Discussion

FcRn expression has long been described as ubiquitous, with no information on its expression. However, in cancer, FcRn is downregulated in some tumor cells and the tumor microenvironment (17, 19, 20, 45), and mechanisms leading to the low FcRn expression are unknown. In this study, we found that autophagy, a well-characterized process in cellular physiology, regulated FcRn levels during macrophage polarization toward the pro- or anti-inflammatory phenotype. We used a minimal model of macrophage differentiation consisting of the addition of GM-CSF or M-CSF to minimize cytokine implication. This model allows for the differentiation of macrophages with typical pro- or anti-inflammatory features (46, 47). The production of cytokines after lipopolysaccharide activation confirmed the differentiated phenotypes of macrophages. Under these conditions, we found FcRn mRNA levels higher in M-Mϕ than GM-Mϕ. This phenotype is acquired as soon as 3 days of differentiation. Moreover, knockdown of FcRn in M-Mϕ deepened the M-Mϕ phenotype and induced a mild reversion of the GM-Mϕ in the M-Mϕ, which confirms the link between FcRn expression and macrophage polarisation subtypes.

The results concerning FcRn mRNA levels in macrophage subtypes agree with transcriptomics data (47). By studying the regulatory networks associated with the GM-CSF or M-CSF signalling pathways, genes contributing to the establishment of an inflammatory program consecutive to monocyte activation were classified. Among the genes differentially transcribed during macrophage polarization with GM-CSF or M-CSF that we identified in Supplementary Data from this study (47), *FCGRT* gene was expressed more in M-Mϕ than monocytes or GM-Mϕ. To deepen this information, we examined FcRn protein expression by flow cytometry and Western blot analysis in our *in vitro* model. We showed an opposite FcRn protein expression as compared to its mRNA levels, with higher FcRn protein levels in GM-Mϕ than M-Mϕ.

Among mechanisms that could explain these data, we explored the autophagy route because of its participation in protein degradation and in cell physiology, especially during monocyte/macrophage differentiation (48). Inhibition of autophagy during this process leads to the blockage of differentiation and impaired phagocytic functions of macrophages (42, 43). Knowing the role of FcRn in phagocytosis (1), the recent participation of *ATG7* autophagy gene in albumin transcytosis in renal tubule epithelial cells *via* FcRn (44) and the well-described FcRn expression in early and late endosomes (13, 14, 49), examining the role of autophagy in FcRn expression seemed obvious. After 6 days of culture, the higher levels of proteins involved in autophagy in M-Mϕ than GM-Mϕ favored greater autophagic capacity and could be responsible for the lower FcRn levels in M-Mϕ than GM-Mϕ. Indeed, early and late endosomes can fuse with autophagosomes to form new complexes called amphisomes (50). Then, amphisomes fuse with lysosomes to lead to protein and organelle degradation/recycling. This phenomenon could then be responsible for FcRn degradation. The high FcRn mRNA levels in M-Mϕ contrasting with the low FcRn protein levels could be explained by a high turn-over of FcRn protein due to its degradation by the autophagic process.

The use of activators and inhibitors of autophagy in our experiments confirmed that FcRn levels depended at least in part on autophagy. This process could occur in parallel to an endoplasmic reticulum-associated degradation of FcRn involving ubiquitin enzymes as described in human cytomegalovirus infection (51). Starvation conditions of culture were more efficient than rapamycin treatment in showing the participation of autophagy in the regulation of FcRn in macrophages. Moreover, chloroquine was of particular interest by showing that when autophagy was inhibited at the autophagosome/lysosome fusion stage, FcRn levels increased in cells. This is clearly seen in GM-Mϕ with high FcRn levels. Moreover, amphisomes can fuse with the plasma membrane before fusion with lysosomes (52). This phenomenon could explain the greater elevated cell-surface levels of FcRn observed in GM-Mϕ than M-Mϕ.

The addition of IgG, one of the main ligands of FcRn, in the culture during macrophage differentiation was also able to modify FcRn expression in macrophages. In M-Mϕ, FcRn and autophagy were both upregulated by IgG. These results could be explained by modifications in FcRn subcellular localization, which is, in this case, engaged in recycling endosomes with IgG protecting against degradation and also by the increase in IgG endocytosis/phagocytosis explaining the high levels of autophagy. In GM-Mϕ, the IVIG addition did not modify FcRn expression probably because of the lower phagocytic capacity of these cells. Recently, Das et al. showed that IVIG induced autophagy in pro-inflammatory but not anti-inflammatory macrophages (53). These discordant results might be explained by the differences in the two models: IVIG was present during the whole culture from day 0 to day 6 and in lower concentrations (up to 100 µg/ml) in our study as compared with the Das et al. study, in which IVIG was added at day 6 in a high concentration (10 mg/ml).

Autophagy participates in the pathophysiology of a wide number of diseases such as inflammatory or autoimmune disorders and cancer. In cancer, autophagy plays a dual role inhibiting benign tumor growth but promoting advanced cancer growth (54). The increased autophagic activity described in aggressive cancers could explain the low FcRn levels found in cancer with poor prognosis (19, 20). Conversely, a defective autophagy process is associated with inflammatory or autoimmune diseases by avoiding the degradation of dysfunctional or cytotoxic cellular components (55, 56), which suggests that modulation of autophagy could be beneficial to ameliorate the diseases. Among treatments applied in these disorders, IVIG is used for its anti-inflammatory effects (57). The mechanisms of action are multiple, in particular the role on the immune response *via* FcγR (58). Modulation of macrophage differentiation toward a pro- or anti-inflammatory immune response with FcRn expression *via* autophagy could account for a new immunomodulatory effect of IVIG.

Altogether, these data implicating autophagy in regulating FcRn expression raise new questions concerning the role of FcRn in immune functions but also in IgG and therapeutic antibody pharmacokinetics and pharmacodynamics. The situation in other cell types expressing FcRn and with or without phagocytic properties needs to be explored to appreciate the importance of such cooperation between autophagy and FcRn.

## Data availability statement

The raw data supporting the conclusions of this article will be made available by the authors, without undue reservation.

## Ethics statement

The studies involving human participants were reviewed and approved by agreement no. CA-REC-2019-188. The patients/participants provided their written informed consent to participate in this study.

## Author contributions

Technical and material support, JL, AL, JM, CD, and M-VD; analysis and interpretation of data, JL and VG-G; visualization, JL; statistical analysis, JL and VG-G; original draft preparation, JL and VG-G; review and editing, VG-G and YD; supervision, VG-G. All authors have read and agreed to the published version of the manuscript.

## Funding

This work was funded by a public grant overseen by the French National Research Agency as part of the *Investissements d’Avenir* program (no. ANR-10-LABX-53-01).

## Acknowledgments

We thank Shawk Karim and Cécile Bergua for their technical help.

## Conflict of interest

The authors declare that the research was conducted in the absence of any commercial or financial relationships that could be construed as a potential conflict of interest.

## Publisher’s note

All claims expressed in this article are solely those of the authors and do not necessarily represent those of their affiliated organizations, or those of the publisher, the editors and the reviewers. Any product that may be evaluated in this article, or claim that may be made by its manufacturer, is not guaranteed or endorsed by the publisher.
