# Supplementary material for: The neonatal Fc receptor expression during macrophage differentiation is related to autophagy
Source: Front Immunol. 2022 Nov 1;13:1054425. doi: 10.3389/fimmu.2022.1054425 (PMC9663809; doi:10.3389/fimmu.2022.1054425)
Supplement: Supplementary file 1 [file DataSheet_1.docx]

Supplementary Material

# Supplementary Figures and Tables

## Supplementary Figure S1


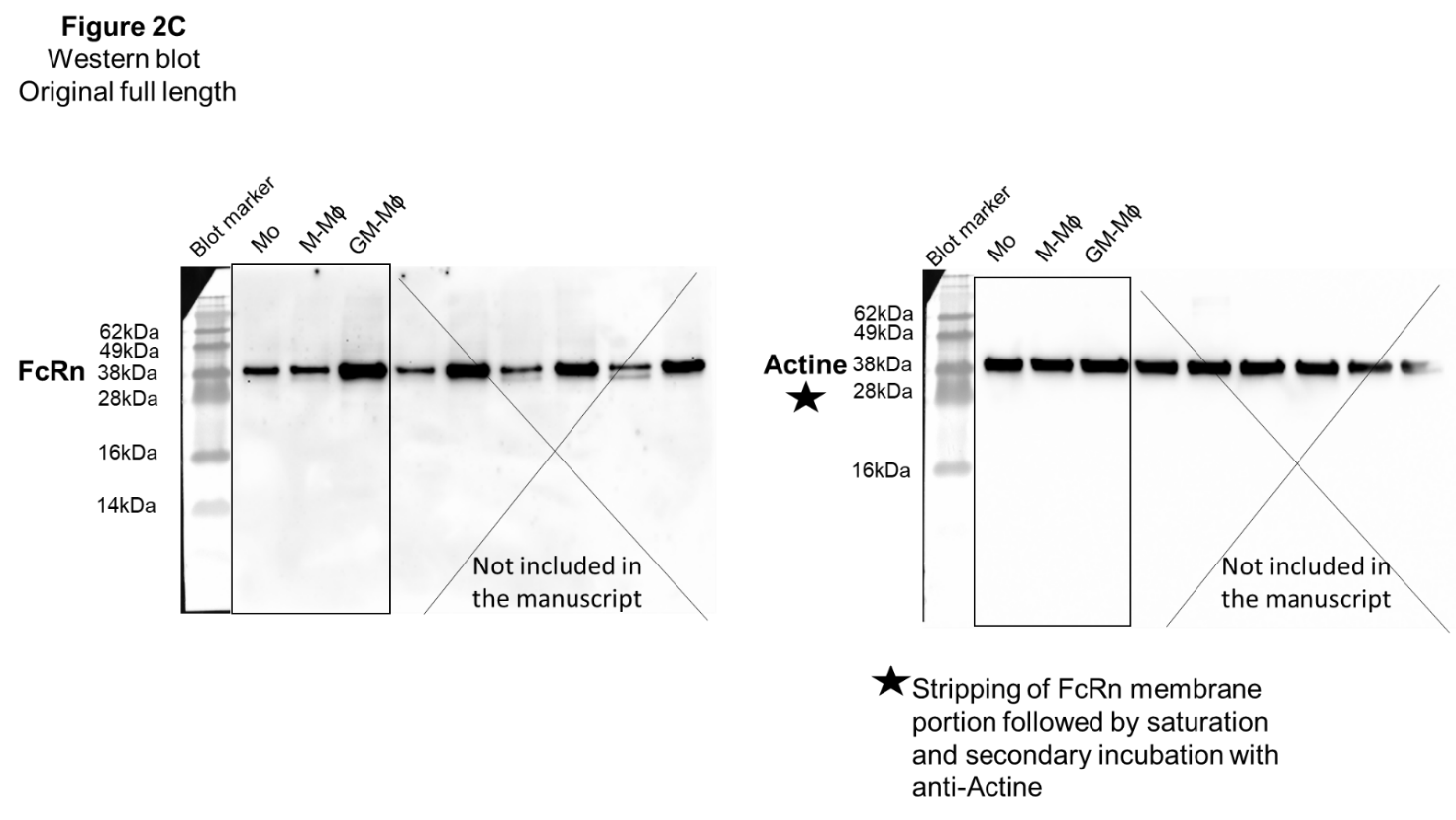


**
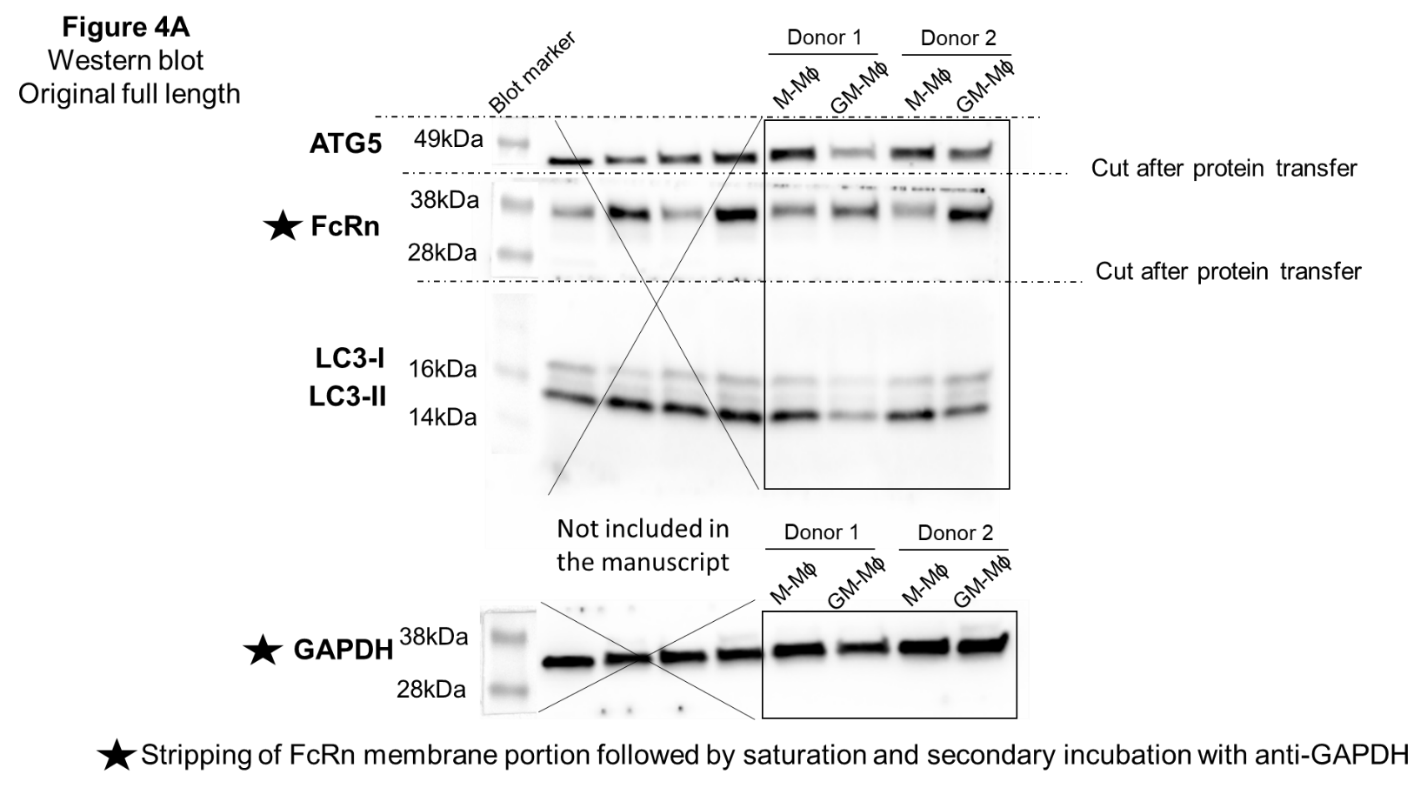
**

**
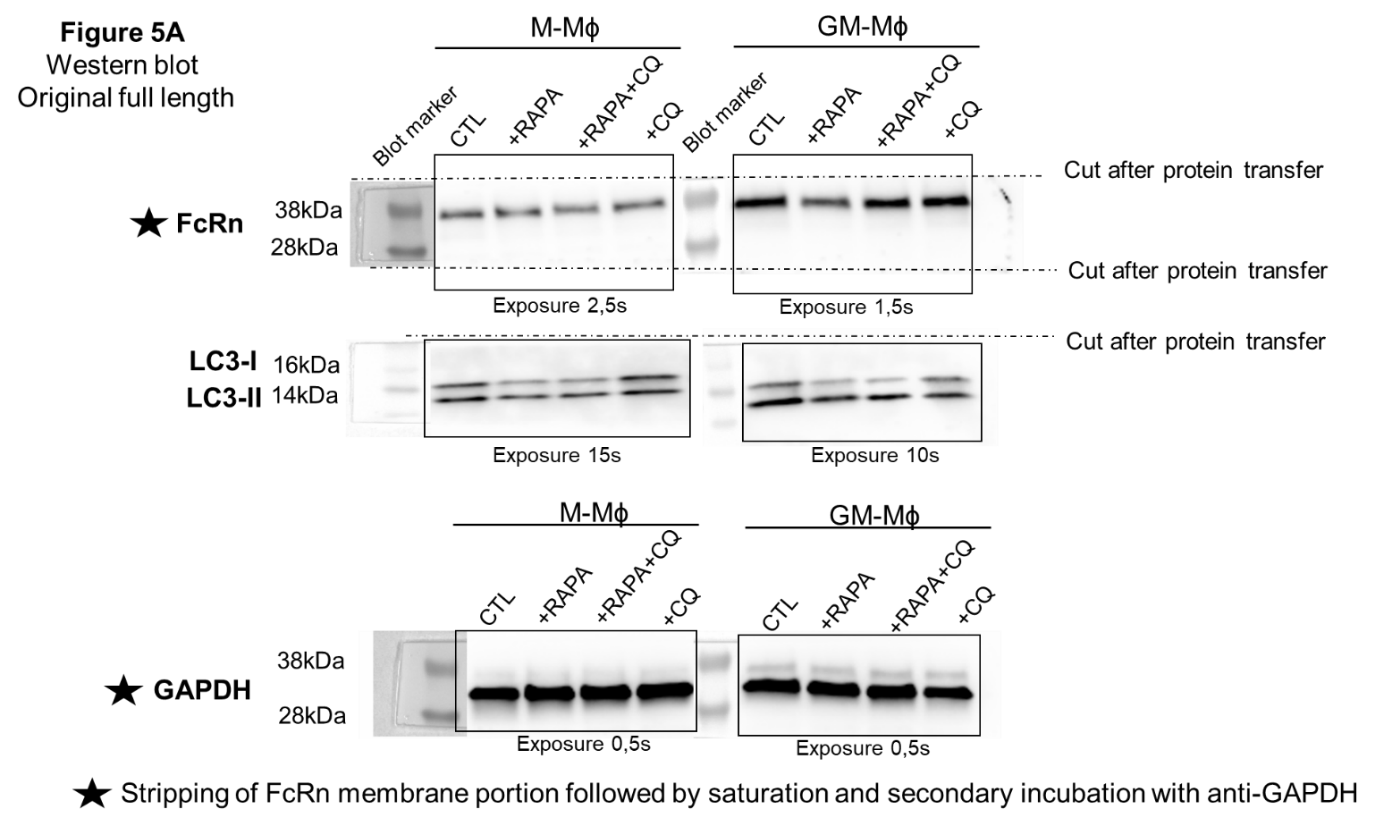
**

**
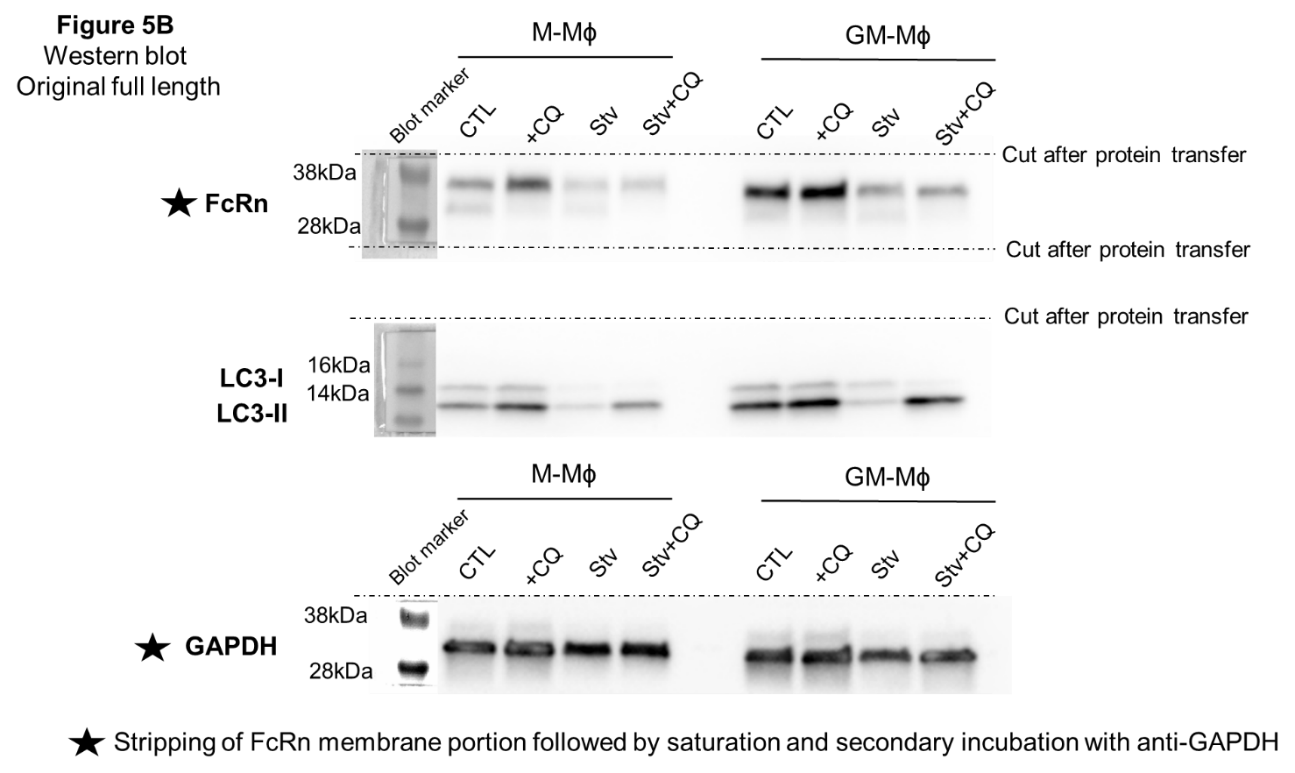
**

**Supplementary Figure S1. Uncropped Western blot raw data from figures 2C, 4A, 5A and 5B.**

##
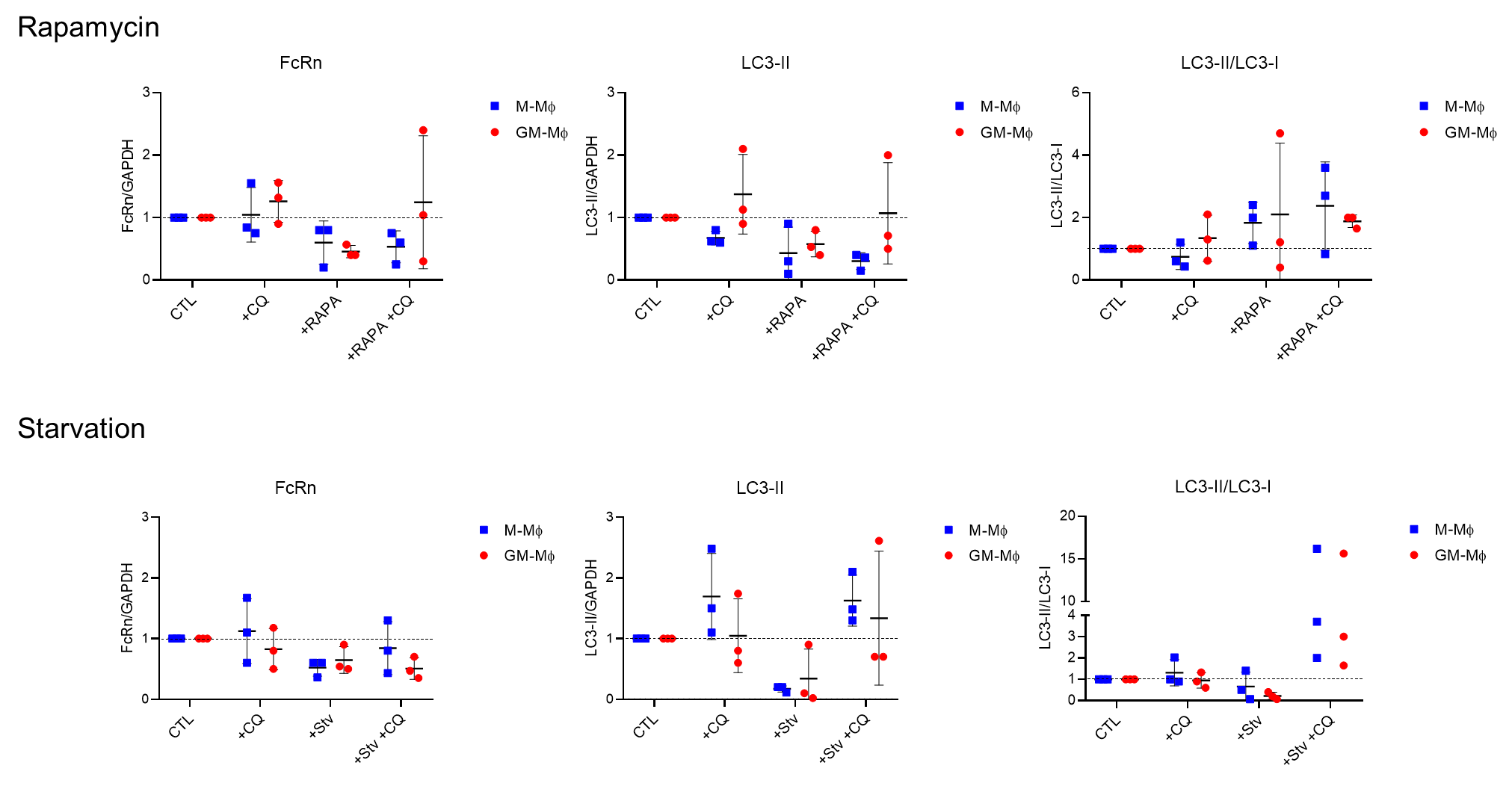
Supplementary Figure S2

**Supplementary Figure S2. Replicates from experiments presented in Figure 5A and 5B (N=3)**

## Supplementary Figure S3


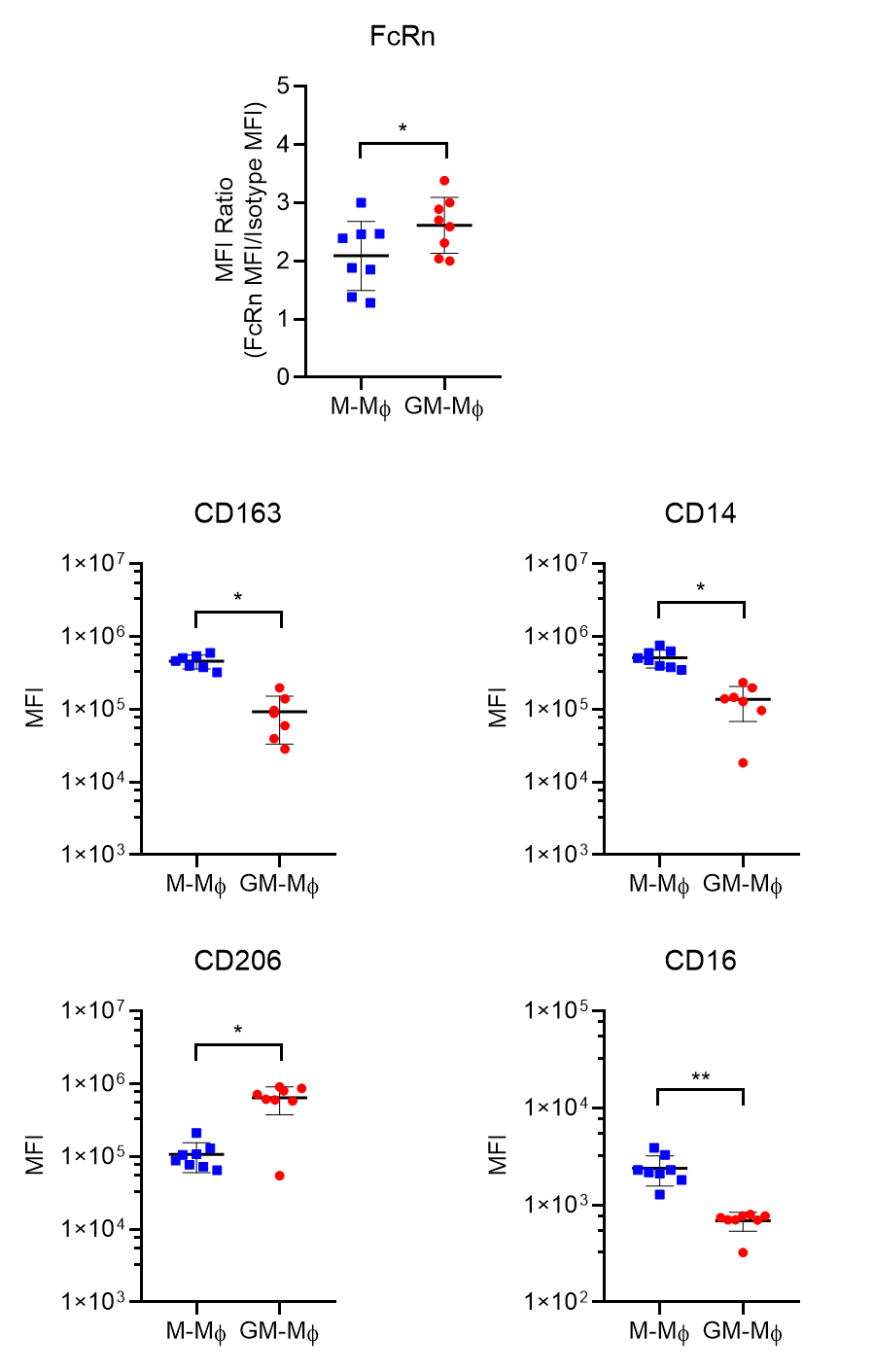

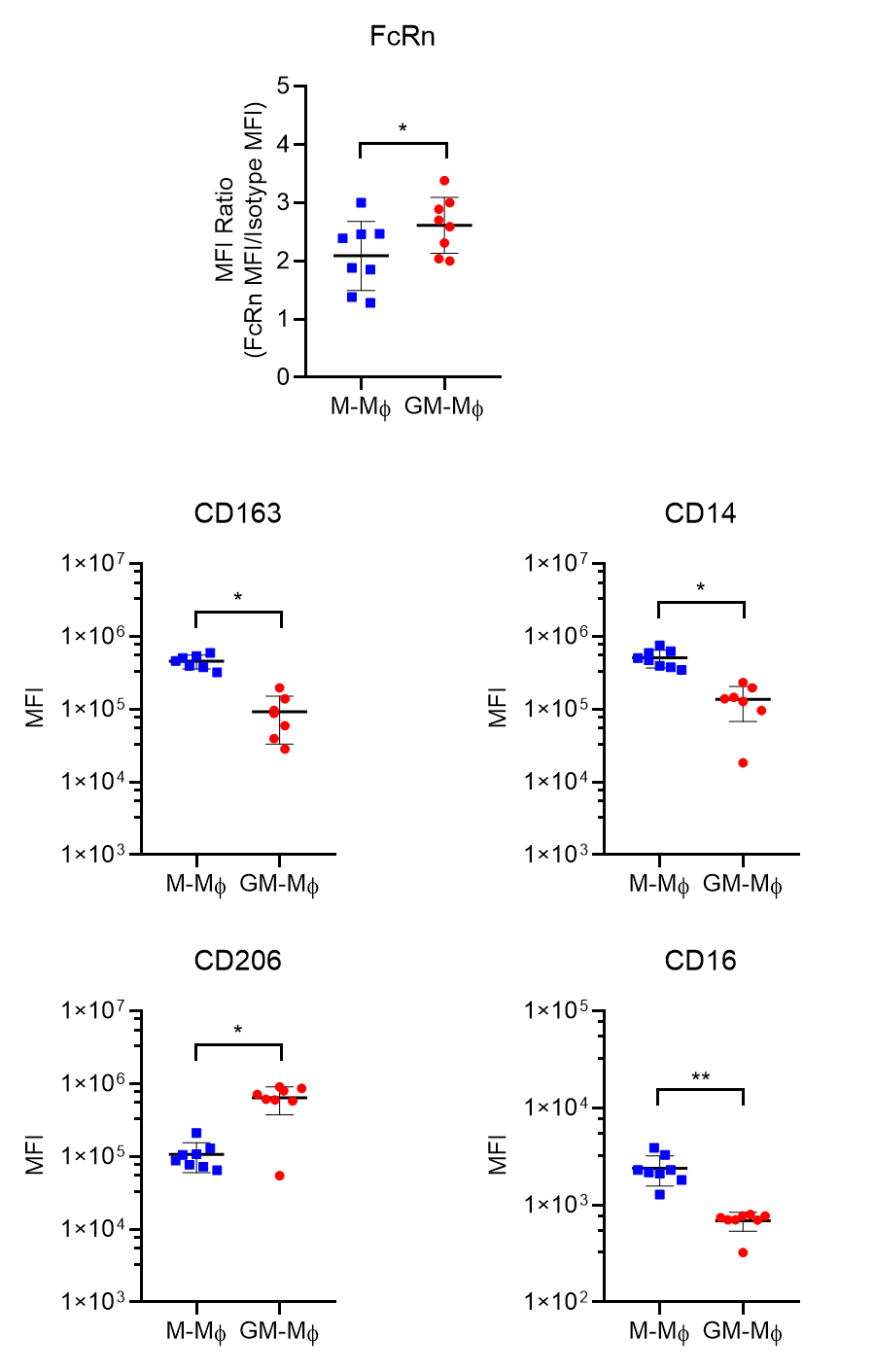
 **A B**

**Supplementary Figure S3.** **Macrophage markers and FcRn analysis by flow cytometry in M-Mϕ and GM-Mϕ at day 3.** Human monocytes were cultured with 60 ng/ml M-CSF or GM-CSF for 3 days. A) CD163, CD14, CD206 and CD16 expression was assessed by flow cytometry on M-Mϕ and GM-Mϕ on day 3. Data are mean MFI (mean ± SD N=8). * p≤0.05 and ** p≤0.01 two-tailed Wilcoxon test. B) Intracellular FcRn expression was analyzed by flow cytometry on M-Mϕ and GM-Mϕ on day 3. Data are mean of MFI ratio between anti-FcRn Ab and isotype (mean ± SD, N=8). Abbreviations: M-Mϕ: M-CSF–induced macrophages, GM-Mϕ: GM-CSF–induced macrophages, MFI, mean fluorescence intensity

## Supplementary Figure S4


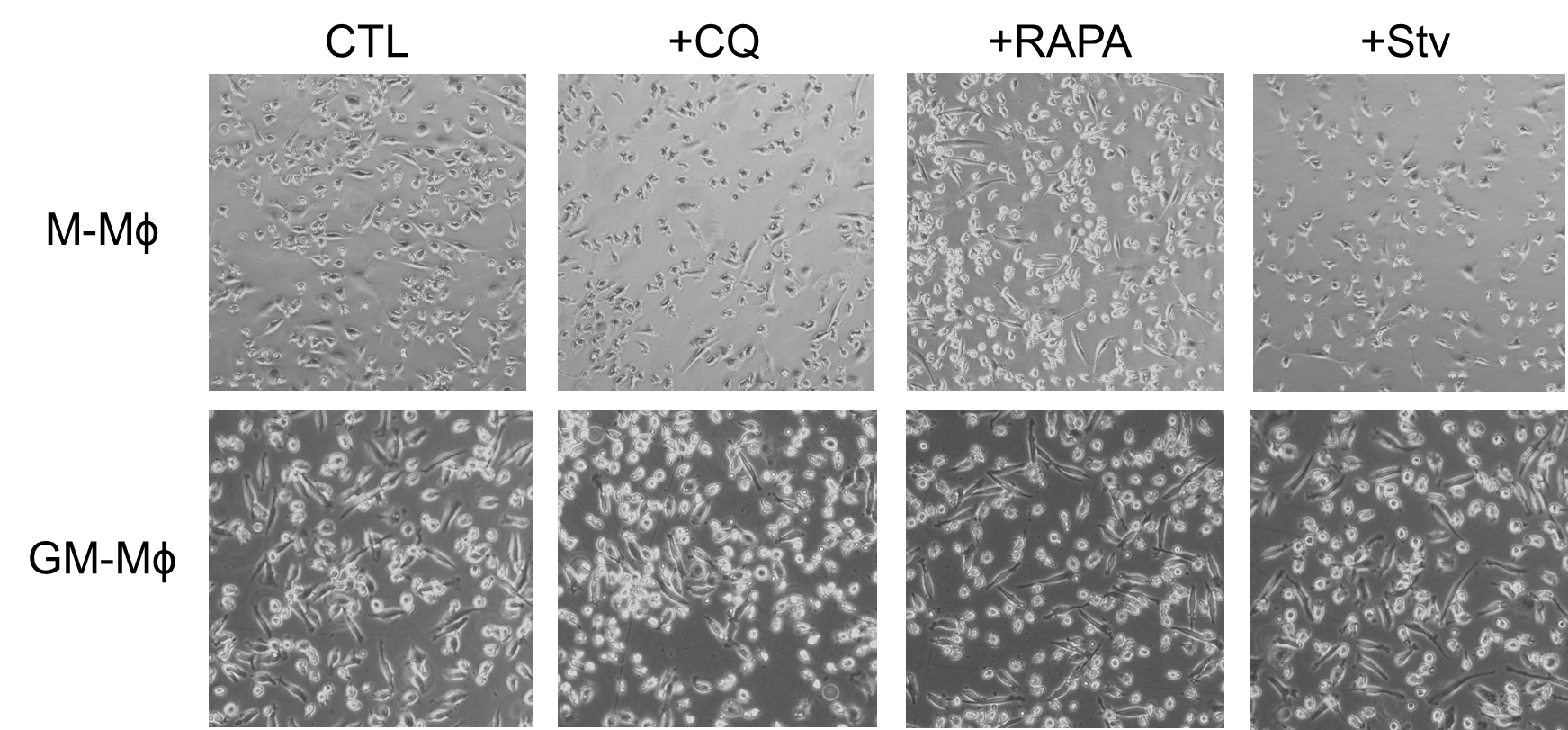


**Supplementary Figure S4. Pictures by photonic microscopy of M- and GM-Mϕ on day 6 following chloroquine, rapamycin and starvation treatment.** M-Mϕ and GM-Mϕ were treated on day 6 with chloroquine for 1 h, on day 5 with rapamycin for 24 h or starved on day 6 for 4 h. Magnification is 100x. Abbreviations: M-Mϕ: M-CSF–induced macrophages, GM-Mϕ: GM-CSF–induced macrophages, CTL: control, RAPA: rapamycin (100 nM), CQ: chloroquine (15 µM), Stv: starvation.

## Supplementary Figure S5

**Supplementary Figure S5. Cell viability analyzed by flow cytometry in M-Mϕ and GM-Mϕ after autophagy induction.** M-Mϕ and GM-Mϕ were treated on day 6 with chloroquine for 1 h, on day 5 with rapamycin for 24 h or starved on day 6 for 4 h. Cell viability was assessed using Annexin V / 7AAD technique (mean ± SD, N=2). Abbreviations: M-Mϕ: M-CSF–induced macrophages, GM-Mϕ: GM-CSF–induced macrophages, CTL: control, RAPA: rapamycin (100 nM), CQ: chloroquine (15 µM), Stv: starvation.

## Supplementary Figure S6

**Supplementary Figure S6. CD16 expression by flow cytometry in M-Mϕ and GM-Mϕ after rapamycin incubation.** M-Mϕ and GM-Mϕ were treated on day 5 with rapamycin for 24h. CD16 expression was assessed by flow cytometry. Data are mean of MFI (mean ± SD, N=4). Abbreviations: M-Mϕ: M-CSF–induced macrophages, GM-Mϕ: GM-CSF–induced macrophages, MFI, mean fluorescence intensity, RAPA: rapamycin
